# Poly[di-μ-aqua-diaqua-di-μ_6_-malonato-cobalt(II)dipotassium(I)]

**DOI:** 10.1107/S1600536811012839

**Published:** 2011-04-16

**Authors:** Adama Sy, Aliou Hamady Barry, Mohamed Gaye, Abdou Salam Sall, Ahmed Driss

**Affiliations:** a Département de Chimie, Faculté des Sciences et Techniques, Université Cheikh Anta Diop, Dakar, Senegal; b Département de Chimie, Faculté des Sciences, Université de Nouakchott, Nouakchott, Mauritania; c Campus Universitaire, Département de Chimie, Faculté des Sciences, Université de Tunis, 1060 Tunis, Tunisia

## Abstract

In the title complex, [CoK_2_(C_3_H_2_O_4_)_2_(H_2_O)_4_]_*n*_, the Co atom is located on a position with site symmetry 2/*m*, the K atom and one water mol­ecule are located on a mirror plane, and the malonate and one water mol­ecule are located on a twofold rotation axis. The K^I^ atom is seven-coordinated by four carboxyl­ate O atoms from four malonate ligands and by three water O atoms, forming a distorted polyhedron. The Co^II^ atom is in an almost octa­hedral environment formed by four carboxyl­ate O atoms from two malonate ligands and two water O atoms. The structure consists of layers parallel to (20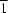) built up from edge-sharing KO_7_ and CoO_6_ polyhedra, which are connected by O—H⋯O hydrogen bonding including water mol­ecules into a three-dimensional network.

## Related literature

For related structures, see: Baggio *et al.* (2003 ▶); Li *et al.* (2004 ▶); Zhao *et al.* (2007 ▶); Wang (2006 ▶).
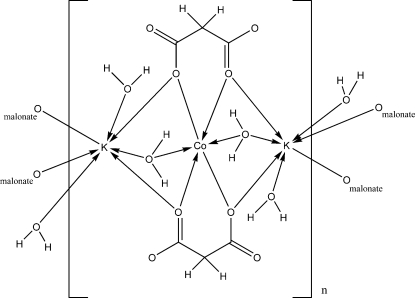


## Experimental

### 

#### Crystal data

[CoK_2_(C_3_H_2_O_4_)_2_(H_2_O)_4_]
*M* *_r_* = 413.28
Monoclinic, 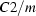

*a* = 9.462 (2) Å
*b* = 11.014 (3) Å
*c* = 7.740 (2) Å
β = 115.65 (2)°
*V* = 727.1 (3) Å^3^
*Z* = 2
Mo *K*α radiation
μ = 1.81 mm^−1^
*T* = 293 K
0.15 × 0.13 × 0.10 mm

#### Data collection

Enraf–Nonius CAD-4 diffractometer
1825 measured reflections
835 independent reflections
813 reflections with *I* > 2σ(*I*)
*R*_int_ = 0.022
Standard reflections: 2; every 120 minutes  intensity decay: none

#### Refinement

*R*[*F* ^2^ > 2σ(*F* ^2^)] = 0.022
*wR*(*F* ^2^) = 0.057
*S* = 1.15
835 reflections
63 parameters
H atoms treated by a mixture of independent and constrained refinement
Δρ_max_ = 0.33 e Å^−3^
Δρ_min_ = −0.30 e Å^−3^

### 

Data collection: *CAD-4 EXPRESS* (Enraf–Nonius, 1994 ▶); cell refinement: *CAD-4 EXPRESS*; data reduction: *MolEN* (Fair, 1990 ▶); program(s) used to solve structure: *SHELXS97* (Sheldrick, 2008 ▶); program(s) used to refine structure: *SHELXL97* (Sheldrick, 2008 ▶); molecular graphics: *ORTEP-3 for Windows* (Farrugia, 1997 ▶); software used to prepare material for publication: *SHELXL97*.

## Supplementary Material

Crystal structure: contains datablocks I, global. DOI: 10.1107/S1600536811012839/bt5510sup1.cif

Structure factors: contains datablocks I. DOI: 10.1107/S1600536811012839/bt5510Isup2.hkl

Additional supplementary materials:  crystallographic information; 3D view; checkCIF report

## Figures and Tables

**Table 1:** Selected bond lengths (Å)

Co1—O2	2.0584 (11)
Co1—O1	2.1347 (18)

**Table 2:** Hydrogen-bond geometry (Å, °)

*D*—H⋯*A*	*D*—H	H⋯*A*	*D*⋯*A*	*D*—H⋯*A*
O1—H*W*1⋯O3^iv^	0.81 (2)	1.91 (2)	2.7077 (17)	167 (2)
O4—H*W*2⋯O3^i^	0.80 (3)	2.03 (3)	2.8372 (18)	176 (3)

Symmetry codes: (i) 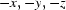; (iv) 
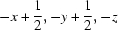
.

**Fig. 1.**
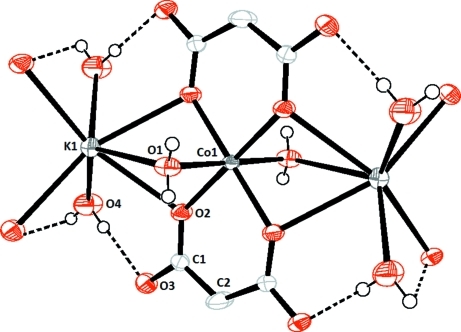
An ORTEP view of the title compound, showing the atom-numbering scheme. Displacement ellipsoids are plotted at the 50% probability level. Broken lines indicate hydrogen bonds.

**Fig. 2.**
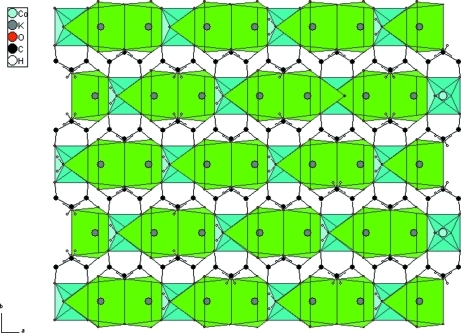
Projection of the structure onto the c axis showing the polyhedra layers connected by the organics molecules.

## supplementary crystallographic information

### Comment

The structure of the title compound is represented in Fig. 1. The Co^II^ cations are in a near perfect octahedral geometry with all *trans*-octahedral angles being 180° and the K ions are all in seven coordinated environment. In the quasi-regular octahedral environment of the six-coordinated Co^II^ cation, basal coordination positions are occupied by four oxygen atoms from two malonate ligands with Co—O distances of 2.0584 (11)Å and the O—Co—O angles in the range [86.32 (5)–93.68 (5) °]. These values agree with those found in literature (Baggio *et al.*, 2003; Li *et al.*). The apical coordination position are occupied by oxygen atoms from water mlecules with bond length [Co—O 2.1347 (18) Å] and an angle value of 180.00 (11) which agree with the values observed for [Co(malonate)(H_2_O]^2-^ cobalt complex (Zhao *et al.*, 2007). The potassium cation has an O7 donor set made up by four µ_2_-bridging malonate oxygen atoms and one µ_2_-bridging water oxygen atoms and two water coordinated molecules. The K cations share four oxygen atoms bridges from malonate groups and two oxygen atoms from water molecule with Co cations. There are hydrogen bonds between water molecules and carbonyl groups of the malonate anions. The cations Co^II^ and K^I^ are arranged in the following sequence: Co—K—K—Co. The metal atoms are found at linear positions [K—Co—K, 180.00 (0)°] as shown in Fig. 2. The Co—K distance is 3.5726 (13) Å. Two K atoms are found to be very close together, having a distance of 4.2086 (14) Å, which is a short metal–metal distance for these types of complexes. The insertion of two polyhedra of KO_7_ between two polyhedra of CoO_6_ results in long Co–Co distances. These two types of geometries form zigzag layers parallel to the ac-plane  and alternating with malonate groups along the *b* axis. The water oxygen atoms provide bridges between K cations. The different polyhedra are still bound to each other through edge-sharing with a compact layer structure defining narrow crossed channels.

### Experimental

In a round bottomed flask, cobalt acetate tetrahydrate (0.4982 g, 2 mmol) dissoveld in a mixture of water and methanol (10 ml, 1:1) was introduced. Imidazole (0.2720 g, 4 mmol) dissolved in 10 ml of the same mixture was added. The solution turn pink. After 10 mn of stirring, 10 ml of a mixture of methanol and water (10 ml, 1:1) containing malonic acid (0.2081 g, 2 mmol) and KOH (0.2240 g, 4 mmol) was added to the pink solution. After 2 h under stirring, the suspension was filtered off and the precipitate was washed with water and diethyl ether before dring under P_2_O_5_. The compound was recrystallized in a mixture of water and dimethylformamide (1/1). After one week, suitable pink crystals for X-ray analyses was obtained. Yield: 72%. m.p. 228±1°C. Anal. Calc. For [C_6_H_12_O_12_K_2_Co]_n_ (%):*C*, 17.44; H, 2.93. Found: C, 17.44; H, 2.93. Selected IR data (cm^-1^, KBr pellet): 3216, 1637, 1600, 1582, 1197, 764.

### Refinement

The H atoms of the water molecules were located in a Fourier difference map and freely refined.  H atoms  of the CH_2_ groups were geometrically placed and refined with a riding model with *U*_iso_(H) = 1.2 *U*_eq_(C).

### Crystal data

**Table:**

[CoK_2_(C_3_H_2_O_4_)_2_(H_2_O)_4_]	*F*(000) = 418
*M**_r_* = 413.28	*D*_x_ = 1.888 Mg m^−^^3^
Monoclinic, *C*2/*m*	Mo *K*α radiation, λ = 0.71073 Å
Hall symbol: -C 2y	Cell parameters from 25 reflections
*a* = 9.462 (2) Å	θ = 11–15°
*b* = 11.014 (3) Å	µ = 1.81 mm^−^^1^
*c* = 7.740 (2) Å	*T* = 293 K
β = 115.65 (2)°	Prism, pink
*V* = 727.1 (3) Å^3^	0.15 × 0.13 × 0.10 mm
*Z* = 2	

### Data collection

**Table:**

Enraf–Nonius CAD-4 diffractometer	*R*_int_ = 0.022
Radiation source: fine-focus sealed tube	θ_max_ = 27.0°, θ_min_ = 2.9°
graphite	*h* = −12→12
ω scans	*k* = −1→14
1825 measured reflections	*l* = −9→9
835 independent reflections	2 standard reflections every 120 min
813 reflections with *I* > 2σ(*I*)	intensity decay: none

### Refinement

**Table:**

Refinement on *F*^2^	Secondary atom site location: difference Fourier map
Least-squares matrix: full	Hydrogen site location: inferred from neighbouring sites
*R*[*F*^2^ > 2σ(*F*^2^)] = 0.022	H atoms treated by a mixture of independent and constrained refinement
*wR*(*F*^2^) = 0.057	*w* = 1/[σ^2^(*F*_o_^2^) + (0.0236*P*)^2^ + 0.6549*P*] where *P* = (*F*_o_^2^ + 2*F*_c_^2^)/3
*S* = 1.15	(Δ/σ)_max_ < 0.001
835 reflections	Δρ_max_ = 0.33 e Å^−^^3^
63 parameters	Δρ_min_ = −0.30 e Å^−^^3^
0 restraints	Extinction correction: *SHELXL97* (Sheldrick, 2008), Fc^*^=kFc[1+0.001xFc^2^λ^3^/sin(2θ)]^-1/4^
Primary atom site location: structure-invariant direct methods	Extinction coefficient: 0.045 (2)

### Special details

**Table:**

Geometry. All e.s.d.'s (except the e.s.d. in the dihedral angle between two l.s. planes) are estimated using the full covariance matrix. The cell e.s.d.'s are taken into account individually in the estimation of e.s.d.'s in distances, angles and torsion angles; correlations between e.s.d.'s in cell parameters are only used when they are defined by crystal symmetry. An approximate (isotropic) treatment of cell e.s.d.'s is used for estimating e.s.d.'s involving l.s. planes.
Refinement. Refinement of *F*^2^ against ALL reflections. The weighted *R*-factor *wR* and goodness of fit *S* are based on *F*^2^, conventional *R*-factors *R* are based on *F*, with *F* set to zero for negative *F*^2^. The threshold expression of *F*^2^ > σ(*F*^2^) is used only for calculating *R*-factors(gt) *etc*. and is not relevant to the choice of reflections for refinement. *R*-factors based on *F*^2^ are statistically about twice as large as those based on *F*, and *R*- factors based on ALL data will be even larger.

### Fractional atomic coordinates and isotropic or equivalent isotropic displacement parameters (Å^2^)

**Table:**

	*x*	*y*	*z*	*U*_iso_*/*U*_eq_	Occ. (<1)
Co1	0.0000	0.0000	0.0000	0.01988 (16)	
K1	−0.27538 (6)	0.0000	−0.49356 (7)	0.03100 (18)	
O1	0.0747 (2)	0.0000	−0.2237 (3)	0.0308 (4)	
O2	0.15473 (12)	0.13440 (10)	0.15312 (16)	0.0290 (3)	
O3	0.24932 (14)	0.31676 (10)	0.26017 (17)	0.0334 (3)	
O4	−0.5000	−0.16509 (19)	−0.5000	0.0479 (5)	
C1	0.14331 (17)	0.24844 (13)	0.1464 (2)	0.0234 (3)	
C2	0.0000	0.3130 (2)	0.0000	0.0519 (8)	
H1	−0.0371	0.3657	0.0720	0.062*	0.50
H2	0.0371	0.3657	−0.0720	0.062*	0.50
HW1	0.134 (2)	0.055 (2)	−0.217 (3)	0.054 (7)*	
HW2	−0.431 (3)	−0.211 (2)	−0.435 (4)	0.066 (8)*	

### Atomic displacement parameters (Å^2^)

**Table:**

	*U*^11^	*U*^22^	*U*^33^	*U*^12^	*U*^13^	*U*^23^
Co1	0.0200 (2)	0.0129 (2)	0.0208 (2)	0.000	0.00322 (16)	0.000
K1	0.0360 (3)	0.0267 (3)	0.0248 (3)	0.000	0.0079 (2)	0.000
O1	0.0357 (9)	0.0200 (7)	0.0416 (10)	0.000	0.0215 (8)	0.000
O2	0.0261 (5)	0.0169 (5)	0.0309 (6)	−0.0021 (4)	0.0001 (4)	0.0004 (4)
O3	0.0328 (6)	0.0228 (6)	0.0330 (6)	−0.0090 (4)	0.0034 (5)	−0.0032 (5)
O4	0.0287 (9)	0.0348 (10)	0.0599 (13)	0.000	0.0000 (9)	0.000
C1	0.0253 (7)	0.0185 (7)	0.0233 (7)	−0.0039 (6)	0.0076 (6)	−0.0002 (5)
C2	0.0451 (15)	0.0169 (11)	0.0555 (17)	0.000	−0.0141 (13)	0.000

### Geometric parameters (Å, °)

**Table:**

Co1—O2^i^	2.0584 (11)	K1—O1^vii^	3.4628 (19)
Co1—O2	2.0584 (11)	K1—K1^iv^	4.2086 (14)
Co1—O2^ii^	2.0584 (11)	K1—HW2	2.89 (3)
Co1—O2^iii^	2.0584 (11)	O1—K1^vii^	3.4628 (19)
Co1—O1	2.1347 (18)	O1—HW1	0.81 (2)
Co1—O1^i^	2.1347 (18)	O2—C1	1.2598 (18)
Co1—K1^i^	3.5726 (13)	O2—K1^i^	2.7987 (13)
Co1—K1	3.5726 (13)	O3—C1	1.2582 (18)
K1—O4	2.7811 (15)	O3—K1^viii^	2.8541 (14)
K1—O4^iv^	2.7811 (15)	O4—K1^iv^	2.7810 (15)
K1—O2^iii^	2.7987 (13)	O4—HW2	0.80 (3)
K1—O2^i^	2.7987 (13)	C1—C2	1.5157 (19)
K1—O3^v^	2.8541 (14)	C2—C1^iii^	1.5157 (19)
K1—O3^vi^	2.8541 (14)	C2—H1	0.9700
K1—O1	3.057 (2)	C2—H2	0.9700
			
O2^i^—Co1—O2	180.00 (11)	O3^v^—K1—O1^vii^	49.62 (3)
O2^i^—Co1—O2^ii^	88.03 (6)	O3^vi^—K1—O1^vii^	49.62 (3)
O2—Co1—O2^ii^	91.97 (6)	O1—K1—O1^vii^	72.74 (6)
O2^i^—Co1—O2^iii^	91.97 (6)	O4—K1—Co1	102.54 (2)
O2—Co1—O2^iii^	88.03 (6)	O4^iv^—K1—Co1	102.54 (2)
O2^ii^—Co1—O2^iii^	180.0	O2^iii^—K1—Co1	35.11 (2)
O2^i^—Co1—O1	86.32 (5)	O2^i^—K1—Co1	35.11 (2)
O2—Co1—O1	93.68 (5)	O3^v^—K1—Co1	118.77 (3)
O2^ii^—Co1—O1	93.68 (5)	O3^vi^—K1—Co1	118.77 (3)
O2^iii^—Co1—O1	86.32 (5)	O1—K1—Co1	36.53 (4)
O2^i^—Co1—O1^i^	93.68 (5)	O1^vii^—K1—Co1	109.27 (4)
O2—Co1—O1^i^	86.32 (5)	O4—K1—K1^iv^	40.83 (3)
O2^ii^—Co1—O1^i^	86.32 (5)	O4^iv^—K1—K1^iv^	40.83 (3)
O2^iii^—Co1—O1^i^	93.68 (5)	O2^iii^—K1—K1^iv^	91.05 (3)
O1—Co1—O1^i^	180.00 (8)	O2^i^—K1—K1^iv^	91.05 (3)
O2^i^—Co1—K1^i^	128.56 (3)	O3^v^—K1—K1^iv^	110.98 (3)
O2—Co1—K1^i^	51.44 (3)	O3^vi^—K1—K1^iv^	110.98 (3)
O2^ii^—Co1—K1^i^	51.44 (3)	O1—K1—K1^iv^	143.21 (4)
O2^iii^—Co1—K1^i^	128.56 (3)	O1^vii^—K1—K1^iv^	144.05 (4)
O1—Co1—K1^i^	121.53 (5)	Co1—K1—K1^iv^	106.68 (3)
O1^i^—Co1—K1^i^	58.47 (5)	O4—K1—HW2	16.2 (5)
O2^i^—Co1—K1	51.44 (3)	O4^iv^—K1—HW2	95.2 (5)
O2—Co1—K1	128.56 (3)	O2^iii^—K1—HW2	107.8 (5)
O2^ii^—Co1—K1	128.56 (3)	O2^i^—K1—HW2	57.0 (5)
O2^iii^—Co1—K1	51.44 (3)	O3^v^—K1—HW2	74.4 (6)
O1—Co1—K1	58.47 (5)	O3^vi^—K1—HW2	150.2 (5)
O1^i^—Co1—K1	121.53 (5)	O1—K1—HW2	111.6 (5)
K1^i^—Co1—K1	180.0	O1^vii^—K1—HW2	123.8 (6)
O4—K1—O4^iv^	81.66 (7)	Co1—K1—HW2	91.0 (5)
O4—K1—O2^iii^	111.08 (4)	K1^iv^—K1—HW2	54.9 (5)
O4^iv^—K1—O2^iii^	70.62 (3)	Co1—O1—K1	85.00 (6)
O4—K1—O2^i^	70.62 (3)	Co1—O1—K1^vii^	167.75 (8)
O4^iv^—K1—O2^i^	111.08 (4)	K1—O1—K1^vii^	107.26 (6)
O2^iii^—K1—O2^i^	63.86 (5)	Co1—O1—HW1	114.1 (18)
O4—K1—O3^v^	78.97 (4)	K1—O1—HW1	124.1 (16)
O4^iv^—K1—O3^v^	137.16 (4)	K1^vii^—O1—HW1	59.3 (17)
O2^iii^—K1—O3^v^	152.22 (4)	C1—O2—Co1	131.78 (10)
O2^i^—K1—O3^v^	97.86 (4)	C1—O2—K1^i^	123.99 (10)
O4—K1—O3^vi^	137.16 (4)	Co1—O2—K1^i^	93.45 (4)
O4^iv^—K1—O3^vi^	78.97 (4)	C1—O3—K1^viii^	128.27 (10)
O2^iii^—K1—O3^vi^	97.86 (4)	K1—O4—K1^iv^	98.34 (7)
O2^i^—K1—O3^vi^	152.22 (4)	K1—O4—HW2	89.5 (19)
O3^v^—K1—O3^vi^	90.00 (5)	K1^iv^—O4—HW2	146 (2)
O4—K1—O1	127.30 (3)	O3—C1—O2	122.62 (14)
O4^iv^—K1—O1	127.30 (3)	O3—C1—C2	115.27 (15)
O2^iii^—K1—O1	58.47 (4)	O2—C1—C2	122.11 (15)
O2^i^—K1—O1	58.47 (4)	C1^iii^—C2—C1	124.1 (2)
O3^v^—K1—O1	94.50 (4)	C1^iii^—C2—H1	106.3
O3^vi^—K1—O1	94.50 (4)	C1—C2—H1	106.3
O4—K1—O1^vii^	127.77 (3)	C1^iii^—C2—H2	106.3
O4^iv^—K1—O1^vii^	127.77 (3)	C1—C2—H2	106.3
O2^iii^—K1—O1^vii^	118.90 (4)	H1—C2—H2	106.4
O2^i^—K1—O1^vii^	118.90 (4)		

Symmetry codes: (i) −*x*, −*y*, −*z*; (ii) *x*, −*y*, *z*; (iii) −*x*, *y*, −*z*; (iv) −*x*−1, −*y*, −*z*−1; (v) *x*−1/2, *y*−1/2, *z*−1; (vi) *x*−1/2, −*y*+1/2, *z*−1; (vii) −*x*, −*y*, −*z*−1; (viii) *x*+1/2, *y*+1/2, *z*+1.

### Hydrogen-bond geometry (Å, °)

**Table:**

*D*—H···*A*	*D*—H	H···*A*	*D*···*A*	*D*—H···*A*
O1—HW1···O3^ix^	0.81 (2)	1.91 (2)	2.7077 (17)	167 (2)
O4—HW2···O3^i^	0.80 (3)	2.03 (3)	2.8372 (18)	176 (3)

Symmetry codes: (ix) −*x*+1/2, −*y*+1/2, −*z*; (i) −*x*, −*y*, −*z*.
